# The diagnostic performance of machine learning-based FFRCT for coronary artery disease: A meta-analysis

**DOI:** 10.1515/med-2025-1320

**Published:** 2025-11-04

**Authors:** Rui Lian, Xiangmin Zhang

**Affiliations:** Department of Gynecology and Obstetrics, West China Second University Hospital, Sichuan University, Chengdu, Sichuan, China; Key Laboratory of Birth Defects and Related Diseases of Women and Children, Ministry of Education, Chengdu, Sichuan, China; Department of Radiology, Children's Hospital of Chongqing Medical University, Chongqing, China; National Clinical Research Center for Child Health and Disorders, Chongqing, China; Ministry of Education Key Laboratory of Child Development and Disorders, Chongqing, China; Chongqing Engineering Research Center of Stem Cell Therapy, Chongqing, China

**Keywords:** machine learning, FFRCT, CAD, meta

## Abstract

**Background:**

This meta-analysis evaluates the diagnostic accuracy of machine learning-derived FFRCT (ML-FFRCT) for CAD, using invasive coronary angiography-derived fractional flow reserve (ICA-FFR) as the gold standard to provide evidence for clinical translation.

**Methods:**

We systematically searched PubMed and Embase for relevant studies. Study quality was assessed using QUADAS-2 in RevMan 5.3. Diagnostic performance was evaluated by pooling sensitivity (SEN), specificity (SPE), positive likelihood ratio (PLR), negative likelihood ratio (NLR), diagnostic odds ratio (DOR), and the area under the curve (AUC) using Stata 14.0. Meta-regression and subgroup analyses were conducted based on the publication year, country, study design, sample source, and sample size.

**Results:**

The pooled SEN was 0.84 (95% CI: 0.79–0.87) and SPE was 0.83 (95% CI: 0.77–0.88). The PLR and NLR were 4.95 (95% CI: 3.58–6.84) and 0.20 (95% CI: 0.15–0.26), respectively. The DOR was 25.15 (95% CI: 14.87–42.52) and the AUC was 0.90 (95% CI: 0.87–0.93), indicating high diagnostic accuracy. Deeks’ funnel plot revealed no significant publication bias.

**Conclusions:**

ML-FFRCT demonstrates high SEN and SPE in diagnosing CAD. These findings support its potential as a promising noninvasive tool for CAD assessment.

## 1 Introduction

Coronary artery disease (CAD), the leading cause of cardiovascular mortality worldwide, is pathologically characterized by atherosclerotic plaque-induced luminal stenosis and myocardial microcirculatory dysfunction [1,2]. Invasive coronary angiography-derived fractional flow reserve (ICA-FFR) remains the gold standard for CAD assessment. This technique measures the ratio of distal coronary pressure to aortic pressure under pharmacologically induced maximal hyperemia, with FFR ≤ 0.80 indicating myocardial ischemia [3]. Despite its diagnostic superiority, the requirements of ICA-FFR for invasive procedures and pharmacologic stress testing significantly increase healthcare costs and procedural complexity, limiting its widespread clinical adoption [4]. Coronary computed tomography angiography (CCTA) has emerged as a preferred noninvasive screening modality due to its rapid imaging acquisition and low radiation exposure [5,6].

However, conventional lumen-based stenosis assessment often overestimates lesion severity, and not all anatomical stenoses cause functional ischemia [7,8]. This “anatomical-functional mismatch” prompted the development of computational fluid dynamics-derived FFR (CFD-FFRCT), which simulates hyperemic hemodynamics using CCTA data to provide a combined anatomical and functional evaluation [9]. Nevertheless, reliance on CFD-FFRCT on complex mathematical modeling results in prolonged computation times, often requiring several hours per case [10]. The advent of machine learning in medical imaging has led to the development of ML-derived FFR (ML-FFRCT), which establishes an ML-derived mapping relationship between CCTA image features and CFD results. Through database training, ML-FFRCT optimizes computational performance and simplifies workstation requirements, delivering objectively quantified hemodynamic assessments within minutes while maintaining diagnostic performance comparable to CFD-FFRCT [11].

Although ML-FFRCT demonstrates significantly improved computational performance over traditional CFD methods, there remains a paucity of systematic evidence evaluating its diagnostic concordance with the gold-standard FFR. This study employs meta-analytic methodology to synthesize the most current research data, assessing the diagnostic efficacy of ML-FFRCT for CAD at the vessel level. Our findings aim to provide evidence-derived support for the clinical translation of this innovative technology and its implementation in precision medicine practice.

## 2 Methods

### 2.1 Literature search strategy

A systematic computerized search was conducted in the EMbase and PubMed databases to identify clinical studies evaluating the diagnostic performance of ML-FFRCT for CAD, published between January 2014 and December 2024. The search was restricted to English-language publications. Two independent researchers performed study selection and data extraction, with any discrepancies resolved by a third investigator. The search strategy combined Medical Subject Headings (MeSH) terms and free-text keywords, including: “Artificial intelligence” OR “Machine learning,” “fractional flow reserve” “FFR” OR “FFRCT,” “CCTA.” Prospero number: CRD420251046889.

### 2.2 Inclusion and exclusion criteria

Inclusion criteria: (1) Publication Type: original clinical studies (in English) evaluating ML-FFRCT for CAD diagnosis; (2) Population: patients who underwent CCTA with AI-derived FFRCT assessment; (3) Reference Standard: invasive FFR (ICA-FFR) as the gold standard, with FFR ≤ 0.80 defining hemodynamically significant ischemia; (4) Data Availability: reported or derivable diagnostic metrics – true positive (TP), false positive (FP), false negative (FN), true negative (TN).

Exclusion criteria: (1) reviews, conference abstracts, case reports (<10 patients), or lacked gold-standard validation; (2) provided insufficient data for meta-analysis calculations; (3) duplicate publications (only the most recent was retained).

### 2.3 Literature screening and data extraction

Two independent investigators (L.R. and Z.X.) performed screening and data extraction using the following protocol: (1) Basic Study Characteristics: author, year, country, study design, sample source, and sample size; (2) Diagnostic Accuracy Data: 2 × 2 contingency tables (TP, FP, FN, TN) and performance metrics (sensitivity, specificity). Discrepancies were resolved via cross-verification, group discussion, or third-party arbitration. Studies with irresolvable ambiguities were excluded. The QUADAS-2 tool (implemented in RevMan 5.3) [12] was used for quality assessment. Disagreements were resolved by consensus.

### 2.4 Statistical analysis

Meta-analysis was performed using Stata 14.0. Heterogeneity Assessment. Heterogeneity was evaluated via Cochran’s *Q* test and quantified using the *I*² statistic, with thresholds defined as follows. 25–49%: low heterogeneity, 50–74%: moderate heterogeneity, ≥75%: high heterogeneity. Random-effects model if *I* ^2^ ≥ 50%, while fixed-effects model if *I* ^2^ < 50%. Diagnostic Performance Metrics Weighted pooled estimates were calculated for pool sensitivity (SEN) and specificity(SPE), diagnostic score and diagnostic odds ratio (DOR), and positive/negative likelihood ratios (PLR/NLR) with 95% CI. A summary receiver operating characteristic curve, with the area under the curve (AUC), was used to quantify overall diagnostic accuracy. Publication bias was assessed using Deeks’ funnel plot asymmetry test. Sensitivity analysis was used to evaluate the impact of individual studies on the overall meta-analysis. Meta-regression and subgroup analysis were stratified by the following study characteristics: publication year (pre-2020 vs post-2020), country (Asia vs non-Asia), study design (retrospective vs prospective), sample source (single-center vs multi-center), and sample size (<200 vs ≥200).

## 3 Results

### 3.1 Studies and selection

**Figure 1:**
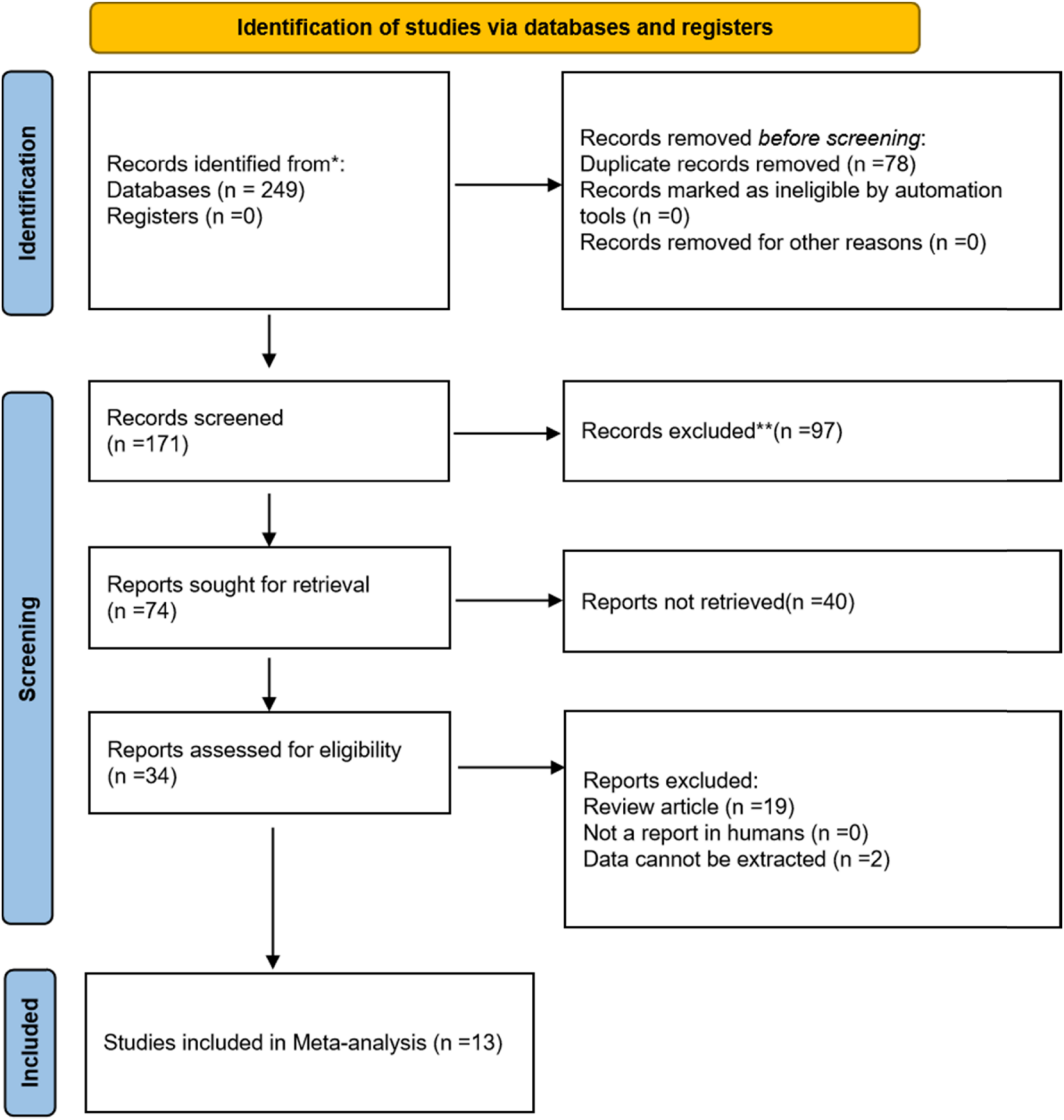
Flowchart of literature search and study selection.

**Figure 2:**
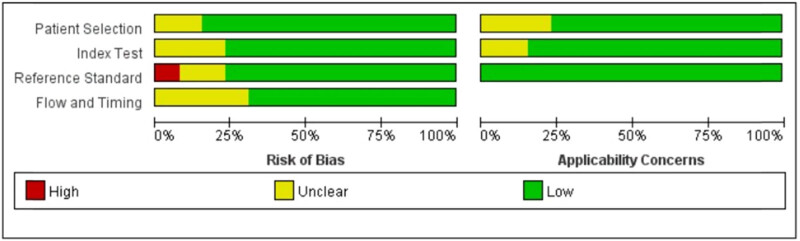
Quality assessment of included studies based on QUADAS-2 tool criteria.

The literature search and screening process are shown in Figure 1, with 249 articles initially identified from PubMed/EMBASE. After removing duplicates and applying inclusion/exclusion criteria, 13 eligible studies [13,14,15,16,17,18,19,20,21,22,23,24,25,26,27] were included. Quality assessment was performed using QUADAS-2 for 13 studies (Figure 2 and Table S1), and most studies showed low risk of bias. As shown in Table 1, the analysis comprised four multicenter and nine single-center studies, predominantly retrospective (ten studies) with only three prospective studies. Geographically, studies originated from Asia (China, Japan, South Korea) and non-Asian countries (Romania, Sweden, Germany, USA, Brazil). Temporally, eight studies were published before 2020 and five studies in 2020 or later. Regarding sample size, 5 studies had ≥200 participants while 8 studies included <200 participants.

**Table 1:** Basic characteristics and diagnostic parameters of the 13 studies

Author	Year	Country	Study design	Sample source	Sample size	Sensitivity	Specificity	TP	FN	TN	FP
Itu [13]	2016	Romania	Retrospective	Single center	125	81.6	83.9	31	7	73	14
Coenen [14]	2018	USA	Prospective	Multi-center	525	81	76	171	41	239	74
Di Jiang [15]	2021	China	Retrospective	Multi-center	544	83	94	161	33	329	21
Yu [16]	2018	China	Retrospective	Single center	166	87.5	78.2	49	7	86	24
Koo [17]	2021	South Korea	Retrospective	Single center	557	63.2	68.1	172	100	194	91
Kurata [18]	2019	Japan	Retrospective	Single center	91	89	75	42	5	33	11
Zhang [19]	2023	China	Retrospective	Multi-center	130	92	85	36	3	77	14
De Geer [20]	2019	Sweden	Retrospective	Multi-center	225	85	72.8	85	15	91	34
Morais [21]	2021	Brazil‌	Retrospective	Single center	152	87	86	65	9	67	11
Li [22]	2019	China	Prospective	Single center	157	88	68	66	9	56	26
Yu [23]	2019	China	Retrospective	Single center	206	81.2	83.6	65	15	105	21
von Knebel Doeberitz [24]	2019	USA	Retrospective	Single center	103	82	94	26	6	67	4
Baumann [25]	2020	Germany	Prospective	Single center	57	87	95	13	2	40	2

TP: true positive value; FP: false positive value; FN: false negative value; TN: true negative value.

### 3.2 Meta-analysis

**Figure 3:**
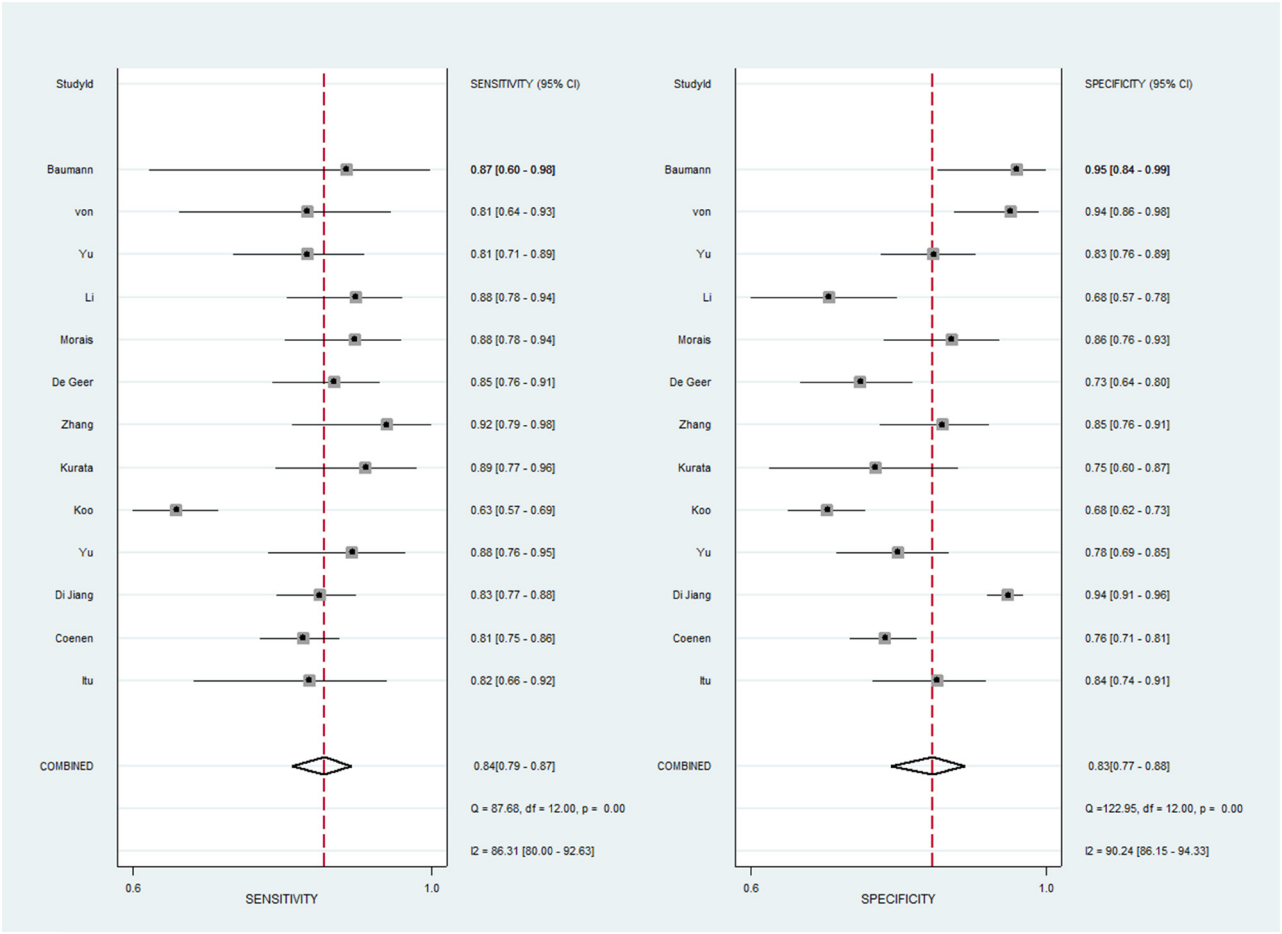
Pool sensitivity and specificity of ML-FFRCT for CAD.

**Figure 4:**
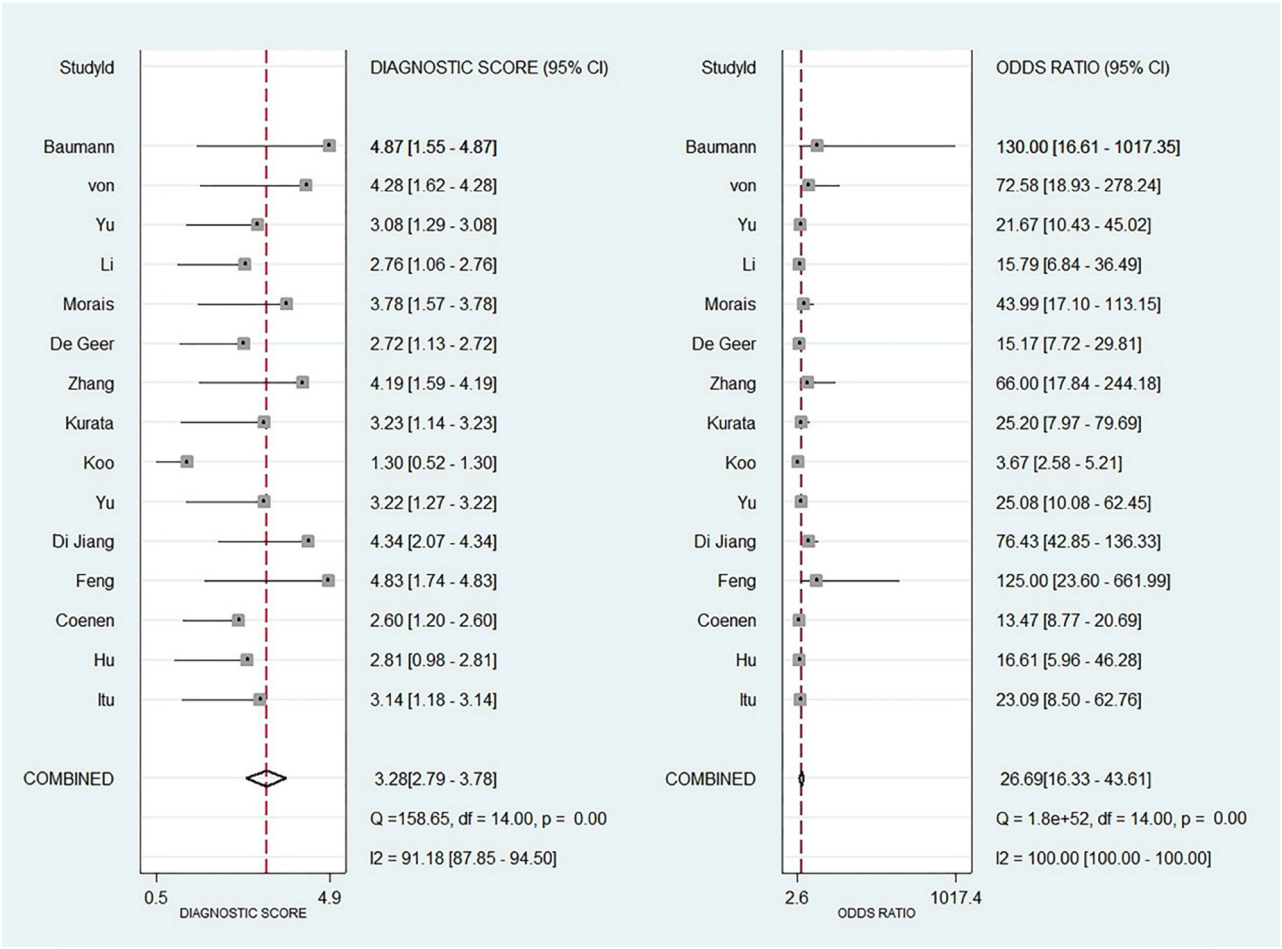
DOR of FFRCT for CAD.

**Figure 5:**
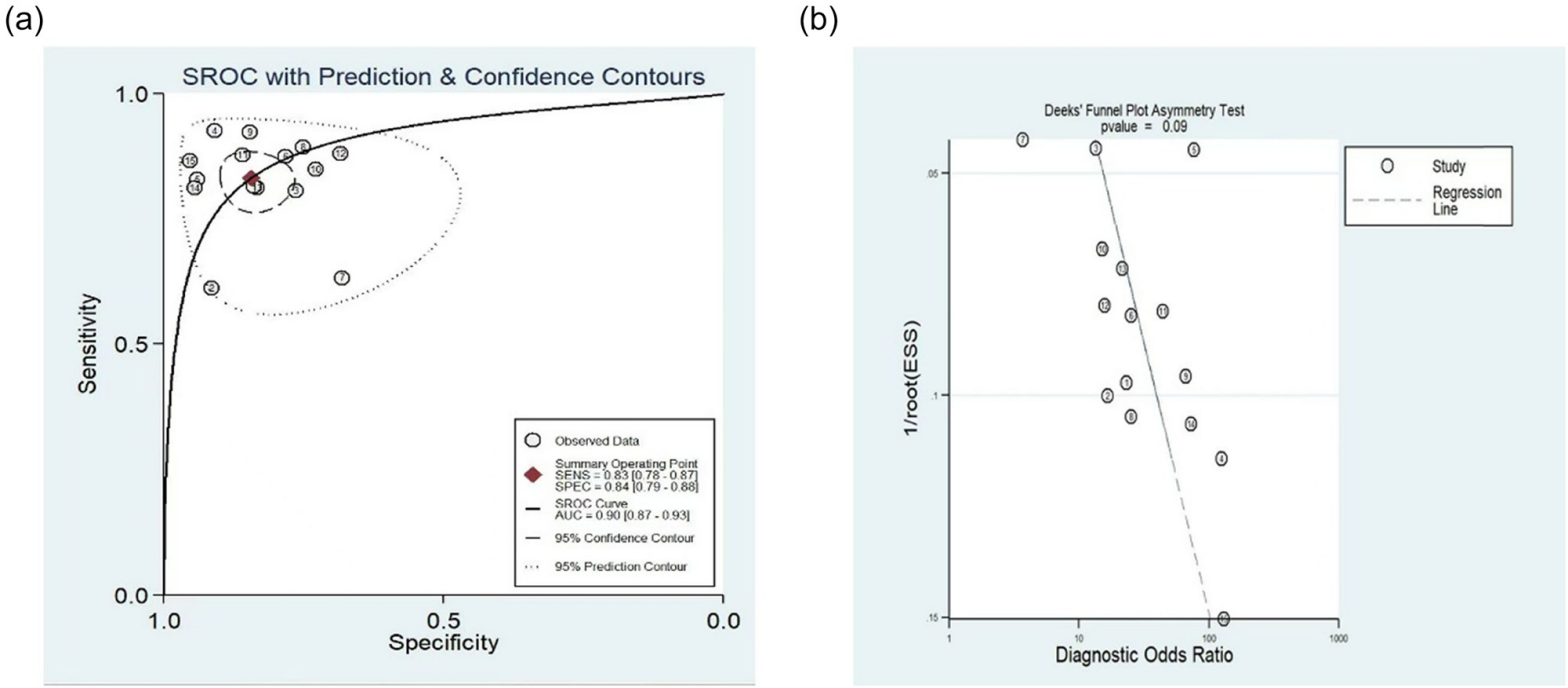
(a) AUC of ML-FFRCT for CAD, and (b) Deeks funnel plot.

**Figure 6:**
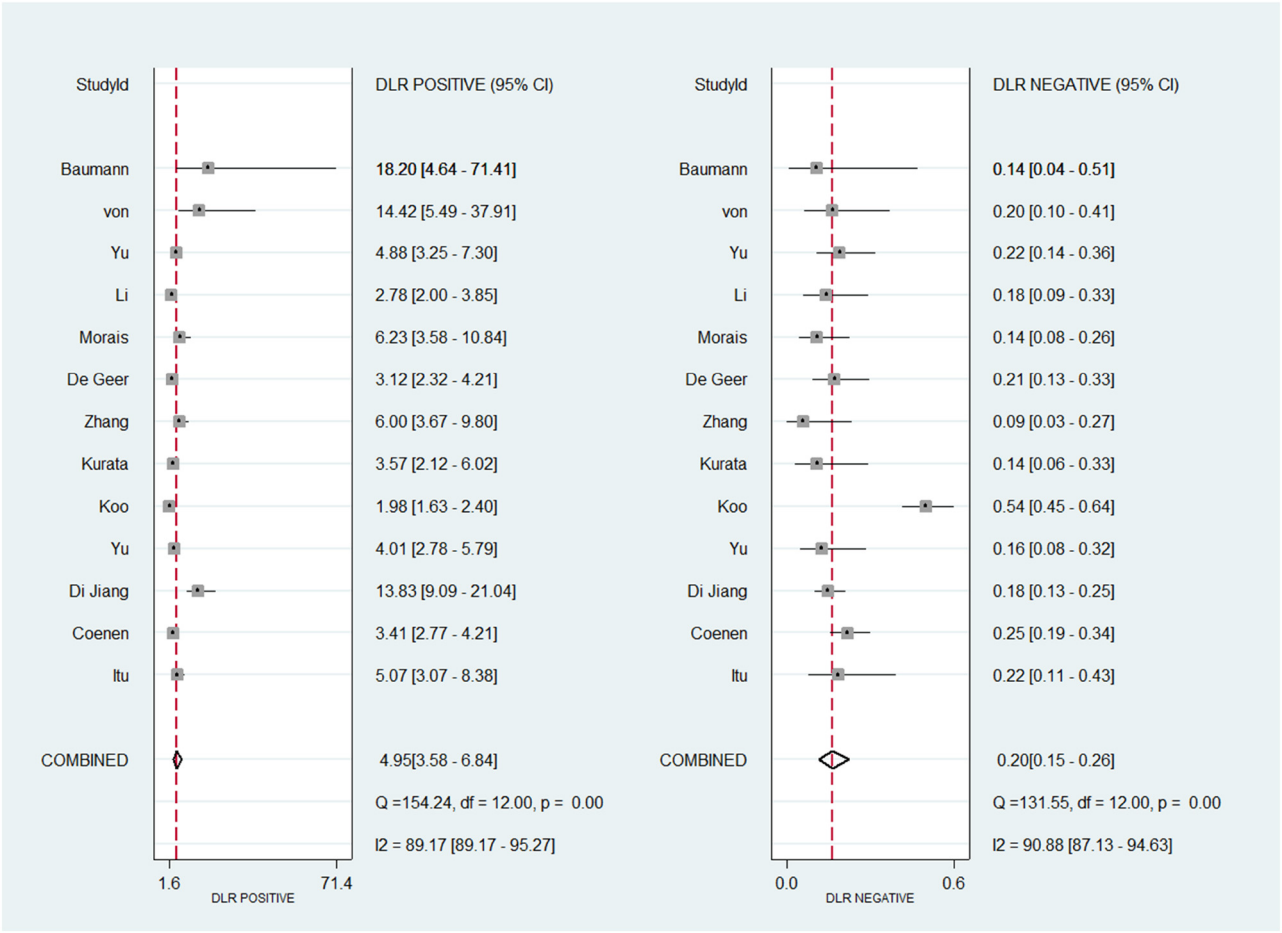
Pool estimates of PLR and NLR.

**Figure 7:**
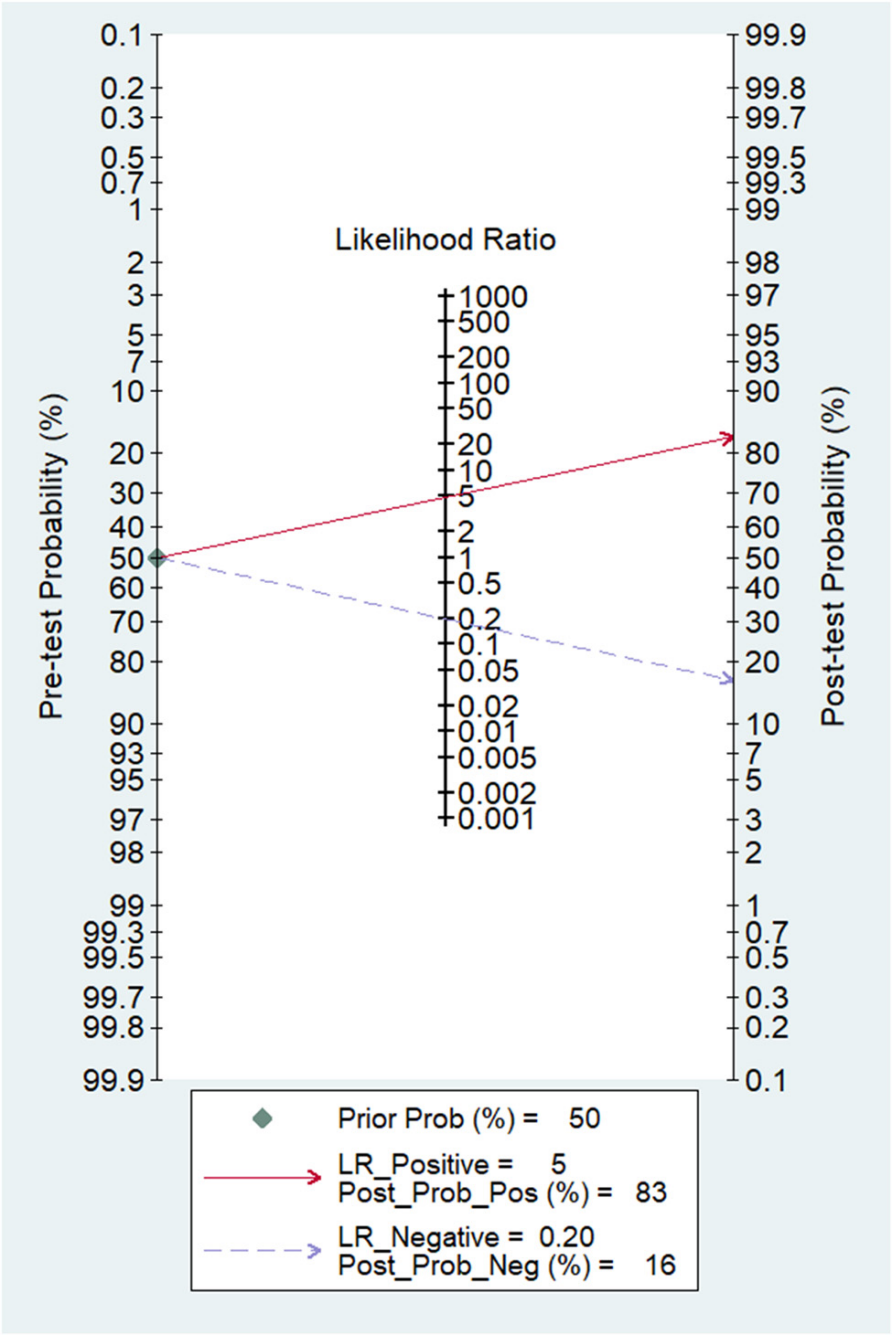
Fagan diagram of ML-FFRCT for CAD.

**Figure 8:**
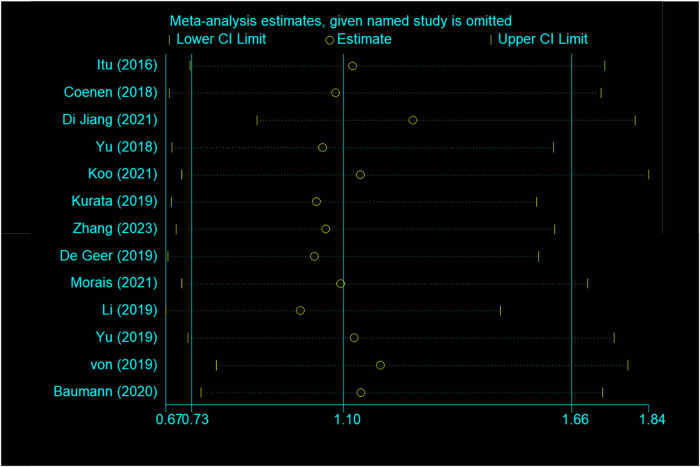
Sensitivity analysis of 13 studies included.

As shown in Figures 3–5, the pooled SEN was 0.84 (95% CI: 0.79–0.87) and SPE was 0.83 (95% CI: 0.77–0.88). The DOR was 25.15 (95% CI: 14.87–42.52) and the AUC was 0.9 (95% CI: 0.87–0.92), demonstrating high diagnostic accuracy for ML-FFRCT. Deeks’ funnel plot revealed symmetrical distribution of studies along the regression line (*p* = 0.15), indicating no significant publication bias. Figure 6 displays PLR of 4.95 (95% CI: 3.58–6.84) and NLR of 0.20 (95% CI: 0.15–0.26), indicating that ML-FFRCT was 4.95 times more likely to correctly identify CAD than misclassify it, while the probability of false negatives was only 0.2 times that of correct exclusions. At a pretest probability of 0.5 (50% CAD prevalence in patients with anatomical stenosis), positive ML-FFRCT results increased diagnostic certainty from 50% to 83%, while negative results reduced misdiagnosis probability to 16% (Figure 7), confirming the strong discriminative capacity of ML-FFRCT (Figure 8).

**Figure 9:**
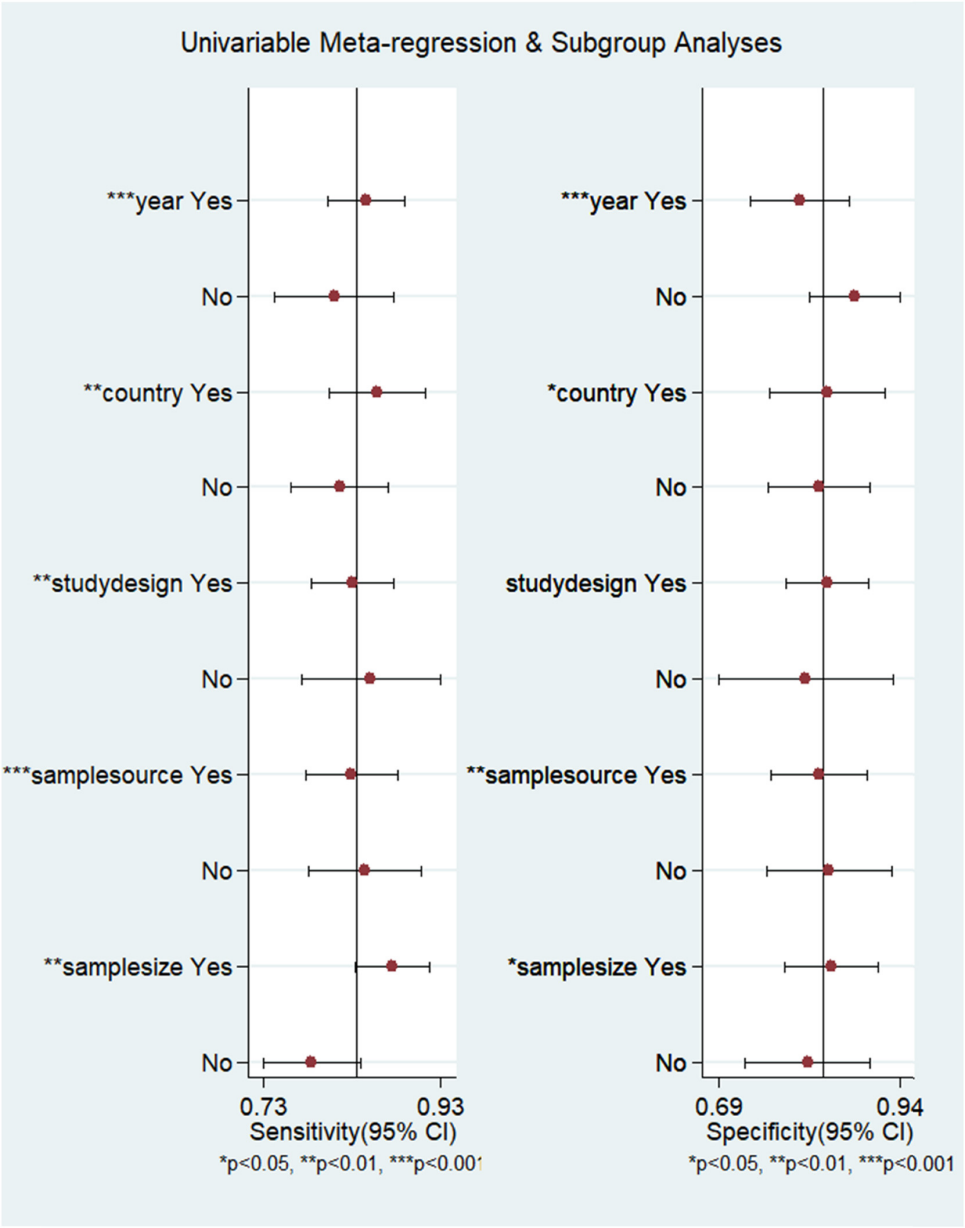
Multiple meta-regression and subgroup analysis on pooled SEN and SPE.

However, significant heterogeneity was observed across all metrics, with *I* ^2^ of 86.31% for SEN, 90.24 for SPE, 89.6% for DOR, 89.17% for PLR, and 90.88% for NLR. The effect sizes of various research reports vary greatly, with DOR ranging from 3.67 to 130. As shown in Figure 9 and Table 2, subgroup analyses and meta-regression identified several significant sources of heterogeneity. For SEN, significant heterogeneity was associated with year (*p* < 0.001), country (*p* < 0.01), study design (*p* < 0.01), sample source (*p* < 0.001), and sample size (*p* < 0.01). For SPE, significant heterogeneity was also observed for year (*p* < 0.001), country (*p* = 0.02), sample source (*p* < 0.01), and sample size (*p* = 0.03), but not for the study design (*p* = 0.11).

**Table 2:** Pooled estimation of subgroups

Subgroup	Number	SEN (95% CI)	SPE (95% CI)	PLR (95% CI)	NLR (95% CI)	DOR (95% CI)	AUC (95% CI)
Before 2020	8	0.83(0.8–0.86)	0.79(0.73–0.84)	4.04(3.13–5.2)	0.21(0.18–0.25)	19.16(13.66–26.89)	0.87(0.84–0.9)
After 2020	5	0.83(0.72–0.9)	0.88(0.78–0.94)	6.76(3.39–13.47)	0.2(0.11–0.35)	34.02(10.46–110.63)	0.92(0.89–0.94)
Asia	8	0.83(0.76–0.88)	0.82(0.75–0.88)	4.71(3.11–7.13)	0.21(0.14–0.31)	22.6(10.57–48.35)	0.89(0.86–0.92)
Non-Asia	5	0.86(0.81–0.89)	0.84(0.74–0.9)	5.27(3.27–8.5)	0.17(0.13–0.22)	30.91(17.57–54.37)	0.89(0.86–0.91)
Retrospective	10	0.83(0.78–0.88)	0.84(0.77–0.88)	5.1(3.58–7.27)	0.2(015–0.27)	25.6(14.06–46.61)	0.9(0.87–0.92)
Prospective	3	0.82(0.76–0.87)	0.81(0.63–0.92)	4.36(2.04–9.33)	0.22(0.15–0.31)	19.92(7.59–52.3)	0.85(0.81–0.87)
Single center	9	0.84(0.77–0.88)	0.82(0.75–0.88)	4.77(3.27–6.96)	0.2(0.14–0.29)	23.92(12.19–46.94)	0.9(0.87–0.92)
Multi-center	4	0.83(0.8–0.86)	0.84(0.73–0.91)	5.19(2.91–9.26)	0.2(0.16–0.25)	25.78(12.32–53.93)	0.84(0.81–0.87)
＜200	8	0.87(0.83–0.9)	0.84(0.77–0.89)	5.51(3.79–8.02)	0.15(0.12–0.2)	35.61(21.7–58.44)	0.9(0.87–0.92)
≥200	5	0.79(0.71–0.85)	0.81(0.7–0.89)	4.17(2.42–7.18)	0.26(0.18–0.38)	15.99(6.61–38.67)	0.86(0.83–0.89)

SEN: sensitivity; SPE: specificity; PLR: positive likelihood ratio; NLR: negative likelihood ratio; DOR: diagnostic odds ratio; AUC: area under the curve; CI: confidence interval.

Despite the observed heterogeneity in country and sample source, the diagnostic performance between Asia and non-Asia, as well as between single-center and multi-center studies, was remarkably consistent, with confidence intervals demonstrating substantial overlap. In terms of study design, retrospective studies showed higher summary estimates for both the DOR (25.6 vs 19.92) and the AUC (0.90 vs 0.85) compared to prospective studies, suggesting a potential overestimation of diagnostic accuracy in retrospective cohorts. Studies conducted after 2020 demonstrated a trend toward superior performance, with notably higher SPE (0.88 vs 0.79), PLR (6.76 vs 4.04), and DOR (34.02 vs 19.16). These findings indicate improved diagnostic accuracy and reliability in recent studies, possibly attributable to advancements in diagnostic techniques, standardized operational procedures, or accumulated investigator experience. Furthermore, studies with a sample size <200 consistently showed elevated summary estimates for SEN (0.87 vs 0.79), DOR (35.61 vs 15.99), and AUC (0.90 vs 0.86) compared to those with larger sample sizes (≥200), with minimal overlap in confidence intervals. This indicates a likely overestimation of diagnostic performance due to small-study bias. Consequently, results from larger studies (sample size ≥200) may provide more reliable and conservative estimates of the true diagnostic accuracy.

## 4 Discussion

This meta-analysis integrating data from 15 studies demonstrates the significant diagnostic performance of ML-FFRCT for CAD, showing a pooled SEN of 0.84, SPE of 0.83, and AUC of 0.90, indicating near-excellent discriminative ability. The DOR of 25.76, along with PLR of 4.95 and NLR of 0.20, confirms the particular advantage of ML-FFRCT in CAD exclusion, which could substantially reduce unnecessary ICA procedures while lowering medical risks and costs. Compared to conventional CCTA relying solely on anatomical assessment, ML-FFRCT overcomes these limitations by employing machine learning to simulate hemodynamics. Our findings highlight the clinical value of ML-FFRCT. Its high SEN minimizes missed diagnoses (especially valuable for atypical CAD presentations), while its strong NLR reliably excludes nonobstructive CAD, preventing overtreatment and making it suitable for both high-risk screening and intermediate/low-risk triage.

CCTA, as a noninvasive anatomical imaging modality, demonstrates unique clinical advantages by ruling out significant stenosis in low-risk patients, making it an ideal screening tool [26]. However, its reliance solely on luminal stenosis assessment may overestimate ischemic risk while potentially leading to unnecessary ICA, revealing inherent functional limitations of anatomical evaluation [27,28]. In functional assessment, ICA-FFR remains the gold standard, particularly valuable for clinically ambiguous borderline lesions [29,30,31], yet carries intrinsic constraints. Its invasive nature risks vascular injury and arrhythmias, while high costs and restricted applicability to revascularization candidates limit screening utility [32]. CFD-FFRCT emerged to address these limitations by solving Navier–Stokes equations to enable completely noninvasive functional assessment, demonstrating superior hemodynamic simulation accuracy in complex anatomies (bifurcation lesions, diffuse stenosis) [33,34]. It can also simulate post-stenting hemodynamic improvements, enabling precise revascularization planning [35]. This technology shows particular specificity for detecting ischemia-causing stenoses, potentially preventing unnecessary interventions and optimizing treatment strategies. However, CFD modeling depends critically on cardiac cycle-dependent coronary flow variations requiring integrated heart-artery modeling, with computational complexity resulting in prolonged processing times (typically hours) [36].

The computational time required for CFD remains a major barrier to its widespread clinical adoption, whereas ML-FFRCT effectively addresses this limitation. Wang et al. [37] demonstrated that AI could automate both geometric quantification of coronary arteries and ML-FFRCT prediction from CCTA images, achieving remarkable sensitivity (97.14%) and specificity (75%) in assessing ischemia risk from stenotic lesions, with a processing time of just 120 ± 13 s. Further studies [38] revealed that machine learning algorithms can automatically extract and analyze key vascular parameters (lumen diameter and cross-sectional area) from both the lesion site and adjacent normal segments for comprehensive evaluation. Compared to CFD, the diagnostic performance of ML-FFRCT is less affected by the “halo effect” of calcified plaques. As machine learning applications in CAD diagnosis continue to mature, these algorithms not only enable sophisticated analysis of CCTA data for noninvasive FFR computation but also, through convolutional neural networks trained on low-dose CT features, can produce coronary image quality comparable to standard-dose acquisitions from reduced radiation scans [39].

However, our analysis revealed substantial heterogeneity with *I*² values exceeding 85%. Such significant heterogeneity may lead to inconsistencies across studies, potentially affecting the robustness of our pooled analysis. Subgroup analyses suggested that year, country, study design, sample source, and sample size might influence the diagnostic performance of ML-FFRCT. Post-2020 studies showed improved specificity and overall reliability, likely reflecting algorithmic and procedural standardization. Most notably, studies with sample sizes ≥200 yielded consistently lower and more conservative estimates of SEN (0.79 vs 0.87), SPE (0.81 vs 0.84), PLR (4.17 vs 5.51), NLR (0.26 vs 0.15), DOR (15.99 vs 35.61), and AUC (0.86 vs 0.90) compared to smaller studies. Smaller studies tend to overestimate effect sizes due to publication bias or methodological variability. Therefore, the subgroup comprising studies with sample sizes ≥200 most likely represents the true diagnostic performance of ML-FFRCT. These larger studies are methodologically more robust, less susceptible to sampling variability and publication bias, and provide more precise and generalizable estimates.

## 5 Limitation

This study provides robust evidence supporting the diagnostic value of ML-FFRCT for CAD. However, several limitations must be acknowledged. Most notably, we observed substantial heterogeneity (*I* ^2^ > 85%). Besides year, country, study design, sample source, and sample size, potential sources of heterogeneity may include differences in specific machine learning algorithms employed, variations in CCTA acquisition protocol, inconsistencies in the application of reference standards, and unmeasured population differences not captured in baseline characteristics. Additionally, over 25% of the included studies were rated as having a potential risk of bias (unclear) in the patient selection domain according to QUADAS-2 assessment, which may represent another source of heterogeneity and potentially impact the validity of the pooled estimates. Although insufficient data were available to perform meta-regression on analyses for these factors, they represent plausible contributors to the variability observed. Furthermore, detailed descriptions of the machine learning algorithms used across included studies were often lacking, limiting further subgroup assessment. The high degree of heterogeneity underscores that our pooled estimates should be interpreted with caution. Clinicians should be aware that the diagnostic performance of ML-FFRCT may vary meaningfully across specific clinical settings and technical implementations.

## 6 Conclusion

Preliminary findings suggest that ML-FFRCT has the potential to serve as a noninvasive adjunct tool with promising diagnostic performance compared to conventional methods. However, its clinical utility must be validated in larger, prospective trials.

## Supplementary Material

Supplementary Table
