# Supplementary material for: The diagnostic performance of machine learning-based FFRCT for coronary artery disease: A meta-analysis
Source: Open Med (Wars). 2025 Nov 4;20(1):20251320. doi: 10.1515/med-2025-1320 (PMC12596863; doi:10.1515/med-2025-1320)
Supplement: Supplementary Table [file med-2025-1320-sm.pdf]

Supplementary material

Table S1: Quality assessment by QUADAS-2

Study	Item	Risk of Bias	Applicability Concerns	Comments
Itu et al. [1]	Patient Selection	◍	◍	*n* = 87, clear inclusion and exclusion criteria.
	Index Test	◍	◍	Operator blind method, standardized processing.
	Reference Standard	◍	◍	Invasive FFR ≤ 0.8.
	Flow and Timing	○		The time interval between CCTA and invasive FFR is unclear.
Coenen et al. [2]	Patient Selection	◍	◍	*n* = 351, clear inclusion and exclusion criteria.
	Index Test	◍	◍	Operator blind method, standardized processing.
	Reference Standard	◍	◍	Invasive FFR ≤ 0.8.
	Flow and Timing	○		The time interval between CCTA and invasive FFR is unclear.
Di Jiang et al. [3]	Patient Selection	◍	◍	*n* = 442, clear inclusion and exclusion criteria.
	Index Test	○	◍	Operator blind method, standardized processing. But contour recognition in calcified lesions still poses challenges.
	Reference Standard	◍	◍	Invasive FFR ≤ 0.8.
	Flow and Timing	◍		The interval between CCTA and invasive FFR is ≤ 90 days.
Yu et al. [4]	Patient Selection	◍	◍	*n* = 129, clear inclusion and exclusion criteria.
	Index Test	◍	◍	Operator blind method, standardized processing.
	Reference Standard	○	◍	Invasive FFR ≤ 0.8, but it did not specify whether it was an independent blind evaluation.
	Flow and Timing	◍		The interval between CTA and invasive FFR is ≤ 2 weeks.
Koo et al. [5]	Patient Selection	○	◍	*n* = 471, however, only including patients with severe CAD may result in selection bias and limited generalizability.
	Index Test	○	○	ML-FFR and CFD-FFR are both based on the same coronary artery segmentation, which not completely blinded.
	Invasive FFR ≤ 0.8.	◍	◍	
	Flow and Timing	◍		The interval between CCTA and invasive FFR is ≤ 90 days.
Kurata et al. [6]	Patient Selection	○	◍	

(*Continued*)

Table S1: *Continued*

Study	Item	Risk of Bias	Applicability Concerns	Comments
				n = 74, but the exclusion criteria are strict, and there may be selection bias.
	Index Test	◍	◍	Operator blind method, standardized processing.
	Reference Standard	◍	◍	Invasive FFR ≤ 0.8.
	Flow and Timing	◍		The interval between CCTA and invasive FFR is ≤ 90 days.
Zhang et al. [7]	Patient Selection	◍	◍	*n* = 462, clear inclusion and exclusion criteria.
	Index Test	◍	◍	Operator blind method, standardized processing.
	Reference Standard	◍	◍	Invasive FFR ≤ 0.8.
	Flow and Timing	◍		The interval between CCTA and invasive FFR is ≤ 90 days.
De Geer et al. [8]	Patient Selection	◍	◍	*n* = 351, clear inclusion and exclusion criteria.
	Index Test	◍	○	Operator blind method, standardized processing. But different KVP settings may have potential impacts
	Reference Standard	◍	◍	Invasive FFR ≤ 0.8.
	Flow and Timing	○		The time interval between CCTA and invasive FFR is unclear
Morais et al. [9]	Patient Selection	◍	○	*n* = 93, only patients suspected of having severe CAD were included
	Index Test	○	◍	standardized processing, but the blind method process is not clear
	Reference Standard	●	◍	Using invasive FFR as the standard, but it is not specified that FFR ≤ 0.8
	Flow and Timing	◍		The interval between CCTA and invasive FFR is < 30 days
Li et al. [10]	Patient Selection	◍	○	Clear inclusion and exclusion criteria. But small sample size, *n* = 45.
	Index Test	◍	◍	Operator blind method, standardized processing.
	Reference Standard	◍	◍	Invasive FFR ≤ 0.8.
	Flow and Timing	◍		The interval between CCTA and invasive FFR is < 2 weeks
Yu et al. [11]	Patient Selection	◍	◍	*n* = 180, clear inclusion and exclusion criteria.
	Index Test	◍	◍	Operator blind method, standardized processing.
	Reference Standard	○	◍	Invasive FFR ≤ 0.8.
	Flow and Timing	◍		The interval between CCTA and invasive FFR is < 2 weeks
Von Knebel Doeberitz et al. [12]	Patient Selection	◍	◍	*n* = 84, clear inclusion and exclusion criteria.
	Index Test	◍	◍	Operator blind method, standardized processing.
	Reference Standard	◍	◍	Invasive FFR ≤ 0.8.

(*Continued*)

Table S1: *Continued*

Study	Item	Risk of Bias	Applicability Concerns	Comments
	Flow and Timing	Low Risk		The interval between CCTA and invasive FFR is ≤ 90 days
Baumann et al. [13]	Patient Selection	Low Risk	Unclear	Clear inclusion and exclusion criteria.But the small sample size (*n* = 40) affects generalization
	Index Test	Low Risk	Low Risk	Operator blind method, standardized processing.
	Reference Standard	Low Risk	Low Risk	Invasive FFR ≤ 0.8.
	Flow and Timing	Unclear		The time interval between CCTA and invasive FFR is unclear.

High Risk. Unclear. Low Risk.

References

[1] Itu L, Rapaka S, Passerini T, Georgescu B, Schwemmer C, Schoebinger M, et al. A machine-learning approach for computation of fractional flow reserve from coronary computed tomography. J Appl Physiol (1985). 2016;121(1):42–52.

[2] Coenen A, Kim YH, Kruk M, Tesche C, De Geer J, Kurata A, et al. Diagnostic accuracy of a machine-learning approach to coronary computed tomographic angiography-based fractional flow reserve: Result from the MACHINE consortium. Circ Cardiovasc Imaging. 2018;11(6):e007217.

[3] Di Jiang M, Zhang XL, Liu H, Tang CX, Li JH, Wang YN, et al. The effect of coronary calcification on diagnostic performance of machine learning-based CT-FFR: A Chinese multicenter study. Eur Radiol. 2021;31(3):1482–93.

[4] Yu M, Lu Z, Li W, Wei M, Yan J, Zhang J. CT morphological index provides incremental value to machine learning based CT-FFR for predicting hemodynamically significant coronary stenosis. Int J Cardiol. 2018;265:256–61.

[5] Koo HJ, Kang JW, Kang SJ, Kweon J, Lee JG, Ahn JM, et al. Impact of coronary calcium score and lesion characteristics on the diagnostic performance of machine-learning-based computed tomography-derived fractional flow reserve. Eur Heart J Cardiovasc Imaging. 2021;22(9):998–1006.

[6] Kurata A, Fukuyama N, Hirai K, Kawaguchi N, Tanabe Y, Okayama H, et al. On-site computed tomography-derived fractional flow reserve using a machine-learning algorithm- clinical effectiveness in a retrospective multicenter cohort. Circ J. 2019;83(7):1563–71.

[7] Zhang XL, Zhang B, Tang CX, Wang YN, Zhang JY, Yu MM, et al. Machine learning based ischemia-specific stenosis prediction: A Chinese multicenter coronary CT angiography study. Eur J Radiol. 2023;168:111133.

[8] De Geer J, Coenen A, Kim YH, Kruk M, Tesche C, Schoepf UJ, et al. Effect of tube voltage on diagnostic performance of fractional flow reserve derived from coronary CT angiography with machine learning: Results from the MACHINE registry. AJR Am J Roentgenol. 2019;213(2):325–31.

[9] Morais TC, Assunção-Jr AN, Dantas Júnior RN, Silva C, Paula CB, Torres RA, et al. Diagnostic performance of a machine learning-based CT-derived FFR in detecting flow-limiting stenosis. Arq Bras Cardiol. 2021;116(6):1091–8.

[10] Li Y, Yu M, Dai X, Lu Z, Shen C, Wang Y, et al. Detection of hemodynamically significant coronary stenosis: CT myocardial perfusion versus machine learning CT fractional flow reserve. Radiology. 2019;293(2):305–14.

[11] Yu M, Lu Z, Shen C, Yan J, Wang Y, Lu B, et al. The best predictor of ischemic coronary stenosis: Subtended myocardial volume, machine learning-based FFR(CT), or high-risk plaque features? Eur Radiol. 2019;29(7):3647–57.

[12] von Knebel Doeberitz PL, De Cecco CN, Schoepf UJ, Duguay TM, Albrecht MH, van Assen M, et al. Coronary CT angiography-derived plaque quantification with artificial intelligence CT fractional flow reserve for the identification of lesion-specific ischemia. Eur Radiol. 2019;29(5):2378–87.

[13] Baumann S, Hirt M, Schoepf UJ, Rutsch M, Tesche C, Renker M, et al. Correlation of machine learning computed tomography-based fractional flow reserve with instantaneous wave free ratio to detect hemodynamically significant coronary stenosis. Clin Res Cardiol. 2020;109(6):735–45.
